# Complete chloroplast genome sequence of *Amomum villosum*

**DOI:** 10.1080/23802359.2019.1644237

**Published:** 2019-07-22

**Authors:** Dong-Mei Li, Chao-Yi Zhao, Gen-Fa Zhu, Ye-Chun Xu

**Affiliations:** Guangdong Key Lab of Ornamental Plant Germplasm Innovation and Utilization, Environmental Horticulture Research Institute, Guangdong Academy of Agricultural Sciences, Guangzhou, China

**Keywords:** *Amomum villosum*, Zingiberaceae, *Amomum*, chloroplast genome, phylogenetic analysis

## Abstract

The first complete chloroplast genome of *Amomum villosum* (Zingiberaceae) was reported in this study. The *A. villosum* genome was 163,608 bp in length, and comprised a pair of inverted repeat (IR) regions of 29,820 bp each, a large single-copy (LSC) region of 88,680 bp, and a small single-copy (SSC) region of 15,288 bp. It encoded 141 genes, including 87 protein-coding genes (79 PCG species), 46 tRNA genes (28 tRNA species), and 8 rRNA genes (4 rRNA species). The overall AT content was 63.92%. Phylogenetic analysis showed that *A. villosum* was closely related to two species *Amomum kravanh* and *Amomum compactum* within the genus *Amomum* in family Zingiberaceae.

*Amomum villosum* is one species of the genus *Amomum* (Zingiberaceae), which distributes predominantly in Fujian, Guangdong, Guangxi and Yunan provinces in China (Wu and Larsen 2000). *Amomum villosum* plants are 1–3 m tall; rhizomes procumbent above ground, clothed with brown, scalelike sheaths; leaves sessile or subsessile; leaf sheath with netlike, depressed squares (Wu and Larsen 2000). Fruits of this species own high medicinal value (Wu et al. 2016). Morphological classification of *Amomum* species is difficult owing to the morphological similarity of vegetative parts among species in genus *Amomum* (Wu and Larsen 2000). Within genus *Amomum*, only two complete chloroplast genomes for species *Amomum kravanh* and *Amomum compactum* have been reported so far (Wu et al. 2017), hindering molecular species identification of *Amomum* species based on chloroplast genomes. Nevertheless, no complete chloroplast genome of *A. villosum* has been reported.

*Amomum villosum* was collected from Banna, Yunnan province, and stored at the resource garden of environmental horticulture research institute (specimen accession no. Av2015), Guangdong Academy of Agricultural Sciences, Guangzhou, China. Total chloroplast DNA was extracted from about 100 g of fresh leaves of *A. villosum* using the sucrose gradient centrifugation method (Li et al. 2012). Chloroplast DNA (accession no. AvDNA2017) was stored at −80 °C in Guangdong Key Lab of Ornamental Plant Germplasm Innovation and Utilization, Environmental Horticulture Research Institute, Guangdong Academy of Agricultural Sciences, Guangzhou, China. Library construction was done using Illumina (Illumina, CA, USA) and PacBio (Novogene, Beijing, China) sequencing, respectively. The Illumina and PacBio sequencing data were deposited in the NCBI sequence read archive under accession numbers SRR8185318 and SRR8184508, respectively. After trimming, 80.8 M clean data of 150 bp paired-end reads and 0.48 M clean data of 8–10 kb subreads were generated. The chloroplast genome of *A. villosum* was assembled and annotated by using the reported methods (Li, Wu, et al. 2019). The annotated complete chloroplast genome sequence of *A. villosum* was submitted to the GenBank (accession no. MK262730).

The complete chloroplast genome of *A. villosum* was 163,608 bp in length, and comprised a pair of inverted repeat (IR) regions of 29,820 bp each, a large single-copy (LSC) region of 88,680 bp and a small single-copy (SSC) region of 15,288 bp. It was predicted to contain a total of 141 genes, including 87 protein-coding genes (79 PCG species), 46 tRNA (28 tRNA species), and 8 rRNA (4 rRNA species). Twenty species genes occurred in double copies, including eight PCG species (*ndhB*, *rpl2*, *rpl23*, *rps7*, *rps12*, *rps19*, *ycf1*, and *ycf2*), eight tRNA species (*trnH-GUG*, *trnI-CAU*, *trnL-CAA*, *trnV-GAC*, *trnL-GAU*, *trnA-UGC*, *trnR-ACG*, and *trnN-GUU*) and all four rRNA species (*rrn4.5*, *rrn5*, *rrn16*, and *rrn23*). All these 20 species genes were located in the IR regions. The *ycf1* gene crossed the bounders of SSC-IRa and SSC-IRb regions, respectively, while the *rps12* gene was located its first exon in the LSC region and other two exons in the IRs regions. In addition, 10 PCG genes (*atpF*, *ndhA*, *ndhB*, *rpoC1*, *petB*, *petD*, *rpl2*, *rpl16*, *rps12*, and *rps16*) and 6 tRNA genes (*trnK-UUU*, *trnG-GCC*, *trnL-UAA*, *trnV-UAC*, *trnI-GAU*, and *trnA-UGC*) had a single intron, while two other genes (*ycf3* and *clpP*) possessed two introns. The nucleotide composition was asymmetric (31.68% A, 18.30% C, 17.78% G, 32.25% T) with an overall AT content of 63.92%. The AT contents of the LSC, SSC, and IR regions were 66.30%, 69.94%, and 58.85%, respectively.

To obtain its phylogenetic position within family Zingiberaceae, a phylogenetic tree was constructed by using single nucleotide polymorphisms (SNPs) arrays from available 15 species chloroplast genomes using *Costus viridis, Costus pulverulentus*, and *Canna indica* as outgroup taxa. The SNP arrays were obtained as a previously described method (Li, Zhao, et al. 2019). For each chloroplast genome, all SNPs were connected in the same order to obtain a sequence in FASTA format. Multiple FASTA format sequences alignments were carried out using ClustalX version 1.81 (Thompson et al. 1997). A maximum-likelihood phylogenetic tree (Figure 1) was constructed using the SNPs from 15 chloroplast genomes alignment result with MEGA7 (Kumar et al. 2016). As shown in the phylogenetic tree (Figure 1), *A. villosum* was closely related to two species *A. kravanh* and *A. compactum* within the genus *Amomum* in family Zingiberaceae with available SNPs.

**Figure 1.**
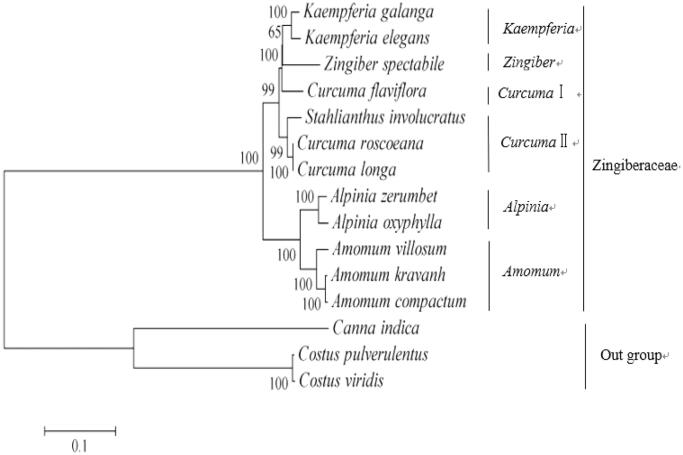
Phylogenetic tree constructed with single nucleotide polymorphisms arrays from 15 species chloroplast genomes using maximum-likelihood method. The bootstrap values were based on 1000 replicates and are indicated next to the branches. Accession numbers: *Alpinia zerumbet* JX088668, *Alpinia oxyphylla* NC_035895.1, *Curcuma flaviflora* KR967361, *Curcuma roscoeana* NC_022928.1, *Curcuma longa* MK262732, *Kaempferia galanga* MK209001, *Kaempferia elegans* MK209002, *Zingiber spectabile* JX088661, *Amomum kravanh* NC_036935.1, *Amomum compactum* NC_036992.1, *Stahlianthus involucratus* MK262725, *Costus pulverulentus* KF601573, *Costus viridis* MK262733, and *Canna indica* KF601570.
